# Flexible, Penetrating Brain Probes Enabled by Advances in Polymer Microfabrication

**DOI:** 10.3390/mi7100180

**Published:** 2016-10-04

**Authors:** Ahuva Weltman, James Yoo, Ellis Meng

**Affiliations:** 1 Department of Biomedical Engineering, University of Southern California, Los Angeles, CA 90089, USA; weltman@usc.edu (A.W.); james.yoo@usc.edu (J.Y.); 2 Ming Hsieh Department of Electrical Engineering, University of Southern California, Los Angeles, CA 90089, USA

**Keywords:** intracortical microelectrodes, brain machine interfaces, polymer neural probes, insertion shuttle

## Abstract

The acquisition of high-fidelity, long-term neural recordings in vivo is critically important to advance neuroscience and brain–machine interfaces. For decades, rigid materials such as metal microwires and micromachined silicon shanks were used as invasive electrophysiological interfaces to neurons, providing either single or multiple electrode recording sites. Extensive research has revealed that such rigid interfaces suffer from gradual recording quality degradation, in part stemming from tissue damage and the ensuing immune response arising from mechanical mismatch between the probe and brain. The development of “soft” neural probes constructed from polymer shanks has been enabled by advancements in microfabrication; this alternative has the potential to mitigate mismatch-related side effects and thus improve the quality of recordings. This review examines soft neural probe materials and their associated microfabrication techniques, the resulting soft neural probes, and their implementation including custom implantation and electrical packaging strategies. The use of soft materials necessitates careful consideration of surgical placement, often requiring the use of additional surgical shuttles or biodegradable coatings that impart temporary stiffness. Investigation of surgical implantation mechanics and histological evidence to support the use of soft probes will be presented. The review concludes with a critical discussion of the remaining technical challenges and future outlook.

## 1. Introduction

**Figure 1:**
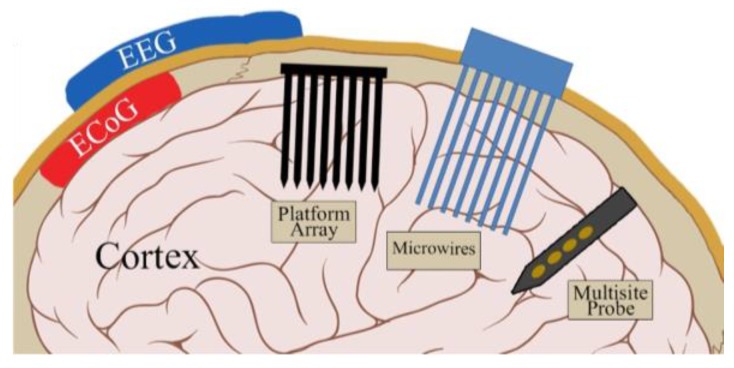
Illustration of the major types of electrode interfaces to the brain: electroencephalography (EEG) are typically discrete electrodes applied to the top of the skull, electrocorticography (ECoG) are electrode arrays supported on a flexible substrate and laid on the surface of the brain, platform array are shanks that penetrate the brain but whose base lies on the surface of the brain, microwires, and multisite probe (where multiple electrodes reside on a single shank). Reproduced with permission [1]. Copyright 2014, John Wiley and Sons.

As neuroscience research evolves, and as more questions about the complexity and functions of the brain arise, so too does the need for more advanced experimental tools. One such tool is a brain–machine interface (BMI) wherein the brain and machine communicate via the common language of electrical activity using electrodes as the communication medium. Arrays of electrodes are produced in a variety of packages and configurations. They can appear as grid-like networks that lie atop the skull, as in electroencephalography (EEG) or atop the brain directly as in electrocorticography (ECoG), and record activity from populations of neurons (see Figure 1) [1]. Alternatively, a penetrating microelectrode can record from a small population of nearby neurons or even a single neuron, enabling precision recording of neural activity, which will be the focus of this review.

**Figure 2:**
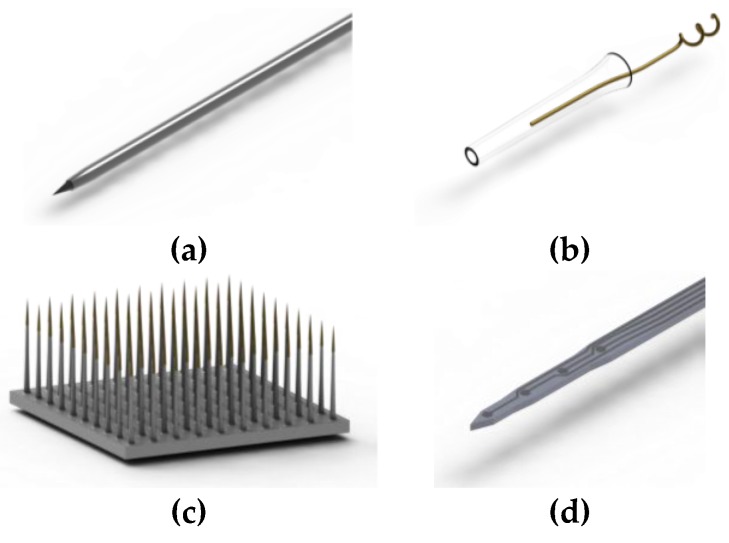
Illustrations of significant rigid probe designs: (**a**) Insulated microwire with exposed tip; (**b**) Glass cone with insulated wire; (**c**) Utah style electrode array; (**d**) Michigan style multi-site probe.

Early penetrating microelectrodes were conductive metal wires that were insulated except at the tip or housed in glass pipettes containing ionic solution (see Figure 2). This limited each probe to a single recording site. With the advent of microelectromechanical systems (MEMS) techniques, batch fabrication of microelectrodes arrays on slender silicon shanks became possible. This allowed for the patterning of multiple electrodes along the length of the probe with creative designs and architectures. For a detailed review of the current state of the field of penetrating intracortical electrodes, including biological and non-biological failure modes and strategies towards improving device performance over time, see these reviews: [2,3]. While there has been a tremendous amount of literature exploring the successes of silicon probes to achieve high-quality neural recordings, one issue that continues to plague the field is that the recording capabilities of these devices wane over time, most failing within a few months. A retrospective evaluation of 78 intracortical, 100-site, Utah Arrays chronically implanted in rhesus monkeys found the average recording lifetime to be 12 months, with a longest successful recording time of 5.75 years [4]. Silicon-based probes thus fall short of the goal to achieve stable, long-term recordings over many years for brain–machine interface technology [5,6]. Implant failure can be associated with a variety of biological (e.g., immune response) and non-biological (e.g., connector or electrode failure) mechanisms, including mechanical damage to or chemical corrosion of electrodes and traces, degradation of passivation layers and insulating coatings, and the foreign body response of the brain to the implant [2]; of these mechanisms, solutions that adequately address the biological immune response are lacking and are critically needed to lengthen the lifetime of neural probes.

Rigid silicon and metal probes suffer inevitable signal degradation over time as chronic tissue inflammation leads to an immune cascade that eventually may wall off of the implant [7,8]. This wall consists of glial cells surrounding the implanted rigid electrodes, which increases the distance between neurons and electrode recording sites resulting in impaired signal-to-noise ratios over time [9]. Whereas metals and silicon have Young’s moduli on the order of hundreds of GPa, the stiffness of brain tissue is orders of magnitudes softer, at around 10^−6^ GPa (see Table 1 for modulus values). This disparity between the stiffness of brain tissue and implantable neural probes is a likely source of tissue damage due to chronic inflammation caused by the natural micromotion of the brain and tethering forces from anchored electrical connections between the probes and brain exterior. It has been proposed that the use of probes fabricated from softer materials can mitigate this damage and attenuate the adverse immune response. In one study, soft poly(p-xylylene) (Parylene) probes were shown to induce only a 12%–17% neuronal loss around the implantation site compared to rigid silicon probes which incurred 40% neuronal loss at four weeks post-implantation [10].

The hypothesis that more compliant probes would cause less microdamage to the surrounding brain area, and therefore limit the attendant foreign body response experienced by the implant, has been the impetus to explore soft polymers as substrates for these devices. The Young’s modulus (E) of rigid silicon devices is 190 and 78 GPa for gold [18], while brain tissue in rats can range from 0.1 to 1.2 MPa (Table 1), resulting in at least five orders of magnitude difference. By switching to polymers, the stiffness mismatch can be reduced. The Young’s modulus of polydimethylsiloxane (PDMS), for example, is on the order of hundreds of kPa, which is very closely matched to the stiffness of brain. This paper will detail the limited evidence available to date that support the use of polymer probes, however it is important to note that much of the data for this review will necessarily be extrapolated from the large body of literature reporting rigid probe data.

One of the first penetrating probes made out of compliant materials was reported in 2001, consisted of a polyimide-based (2.3–8.5 GPa) three probe array inserted into the rat barrel cortex, and achieved acute recordings with a maximum signal to noise ratio of 5:1 [19,20]. This proof-of-concept experiment was motivated by the desire to achieve movement of the implant in sync with the brain during natural micromotion (e.g., arising from cardiac pulse or respiration), thereby ameliorating damage to surrounding tissue. The idea that compliant substrates could attenuate probe micromotion—the relative movement between implant and brain, or, for tethered implants, the movement between the brain and skull which can then propagate to the implant-brain interface—was first documented in reference to polyimide based peripheral electrodes and retinal arrays [21,22]. These early reports have been followed by a large body of research seeking to achieve flexible penetrating neural probes suitable for basic neuroscience and prosthetic research, including the exploration of new soft, flexible materials.

A range of studies, from in vitro and in vivo to modeling studies, have examined the effect of implant stiffness modulation on surrounding brain tissue, providing evidence to support the use of softer substrates in brain probes.

3D finite-element modeling work comparing silicon (200 GPa), polyimide (3 GPa), and soft probes (with an elastic modulus of 6 MPa, made out of a hypothetical material) showed that polyimide provided strain relief against tangential tethering forces (probe displacing in *z*-direction), but that soft probes provided relief against both tangential and radial (*x*-*y* displacement) forces [23]. Flexible probes were theorized to absorb micromotion forces mostly in the length of probe proximal to the point of tethering, thereby minimizing the magnitude of forces that interfered with the tip of the probe. This same study indicated that soft probes could reduce interfacial strains by up to 94% during vertical displacement, as compared to silicon counterparts. Benchtop modeling studies used gelatin (15 kPa) as a brain model and compared flexible microelectrode arrays of polyimide to rigid microwire implants. The major findings were that bending, mechanical shock, and lateral deflection tests, which simulated shifting of the brain within the cranium, caused tearing and disruption of the gelatin matrix only by rigid implants, while the flexible implants imparted no damage beyond the initial tract formed during implantation [21].

In vitro studies further support the use for soft materials, indicating that softer substrates are more effective promoters of cell growth than their stiff counterparts. Neurons grown on bisacrylamide gel with a stiffness of 0.23 MPa were shown to grow three times more branches than those grown on a gel with twice the stiffness [24]. Dorsal root ganglion neurite extension growth rate on agarose was reduced by more than half as the stiffness of agarose increased from 0.75% to 2% (wt/vol) concentrations [25], corresponding to moduli from ~10 to 200 kPa, respectively [26].

The impact of substrate stiffness on glial cells, including microglia and astrocytes, was examined as well. Cells plated on either a stiff (100 kPa) or soft (10–30 kPa) substrate exhibited changes in cell morphology and protein expression that were similar to those typically observed in in vivo immune responses. Glial cells on the stiffer substrate developed more expansive and denser cellular processes than their counterparts grown on the softer substrate. This behavior is reminiscent of the physical shape of glial cells, the main immune players, when activated. Cells grown on stiffer substrate were also shown to upregulate inflammatory mediators toll-like receptor 4 (TLR4) and peroxisome proliferator-activated receptor γ (PPARγ). Likewise, acute in vivo studies involving immunohistochemical staining around implants in rat brains revealed that cells neighboring stiffer areas of the implant exhibited elevated inflammatory marker CD11b after just one week of implantation and increased glial fibrillary acidic protein (GFAP) levels after three weeks of implantation. Elevated GFAP levels indicate the presence of activated astrocytic cells, which suggests that mechanical mismatch between electrodes and nervous tissue may enhance unfavorable tissue reactions to the implant that impair its performance [27].

Finally, there are chronic data from in vivo studies to support these claims as well. A study of compliant poly(vinyl acetate) rat brain implants with Young’s moduli in the tens of MPa, compared to a five-times stiffer, chemically matched silicon control showed a comparatively reduced pro-inflammatory cytokine Iba1 and CD68 release around a 200-µm radius from compliant probes at 2, 8, and 16 weeks post-implantation. By 16 weeks post-implantation, NeuN staining for neuronal density revealed a return to normal around compliant probes but not for silicon implants [28]. This suggests the importance of the role of scarring as well as subsequent local neurodegeneration that adversely affect recording quality [29]. The stability of the blood-brain barrier was assessed by IgG infiltration tests, revealing less damage was caused by compliant probes.

It is interesting to consider where exactly the defining line between materials considered to be rigid and materials labelled soft or compliant actually falls. Most materials categorized as compliant, such as polydimethylsiloxane (PDMS), poly(p-xylylene) (Parylene), and polyimide, have Young’s moduli in the tens of GPa, compared to silicon, metals, and ceramics whose Young’s moduli are at least an order of magnitude larger. It is also important to note that the thinner a material is made, the more compliant it will be. Even thinned silicon can exhibit flexible properties. For the scope of this paper, however, “soft” probes will be defined as those made of polymeric substrates with Young’s moduli of 10 GPa or less.

The creation of neural probes out of “soft” materials comes is accompanied by several design limitations, some of which are specific to flexible neural probes, and others which affect the design of neural probes regardless of their stiffness. General trade-offs include those relevant to the density of neural shanks in probe arrays as well as size constraints on electrodes. A goal of neural probe technology is to achieve long-term, stable, single unit recordings from the brain target of interest. While it is clear that increased density of electrical sites is required to achieve this goal, probe shanks can only be packed so closely together before physically displacing the majority of the brain tissue between each shank, making recording or stimulation an impossibility. As electrodes with smaller and smaller surface areas are fabricated in the hopes of targeting and discerning individual neurons, this decrease in surface area can cause large increases in electrode impedances, thereby limiting their functionality. These are some of the design limitations that plague rigid and soft neural probes alike. While soft probe flexibility can be advantageous in limiting the immune response to the implant over time, it complicates the surgical insertion of the probe. Many designs of flexible probes are unable to penetrate brain tissue on their own without buckling, and require the use of temporary or permanent insertion aids to assist in the correct placement of these probes at the target of interest. However, these insertion aids can be bulky, adding to the acute damage incurred to the brain tissue during insertion, and the use of a permanent, stiff, insertion aid can defeat the purpose of using flexible substrates in the first place. This review paper sets out to provide a detailed summary of insertion methods available for the proper placement of flexible neural probes.

Given their mechanical advantage, this review specifically examines recent progress in polymer-based, “soft”, penetrating neural implants. This review will focus solely on electrical interfaces that penetrate the brain. We begin by discussing probe fabrication and material choice. We then transition to design considerations of interest for creating long-term brain interfaces with penetrating probes. As previously discussed, many of the suggested designs and insertion requirements are extrapolated from data of rigid probe studies. To date, the number of chronic studies of polymer probe recordings remains very limited; flexible probes are more commonly evaluated under acute settings and histological assessments of their compatibility with brain tissue is not often performed on functioning, recording electrodes. The only literature source for a chronic study of electrode recording viability on flexible substrates, to the knowledge of these authors, is that performed by Sohal, which lasted for 678 days in rabbits [30]. Anatomical requirements are captured in a discussion on mechanical constraints—namely tissue hardness, insertion depth, and probe configuration. In particular, a number of customized insertion techniques will be discussed including consideration of the speed of implantation. To realize practical probe systems, electronic packaging methods and strategies are explored. The review concludes by examining the remaining technical obstacles and future prospects of flexible intracortical probes.

## 2. Probe Fabrication and Material Selection

### 2.1. Basics of Microfabrication

**Figure 3:**
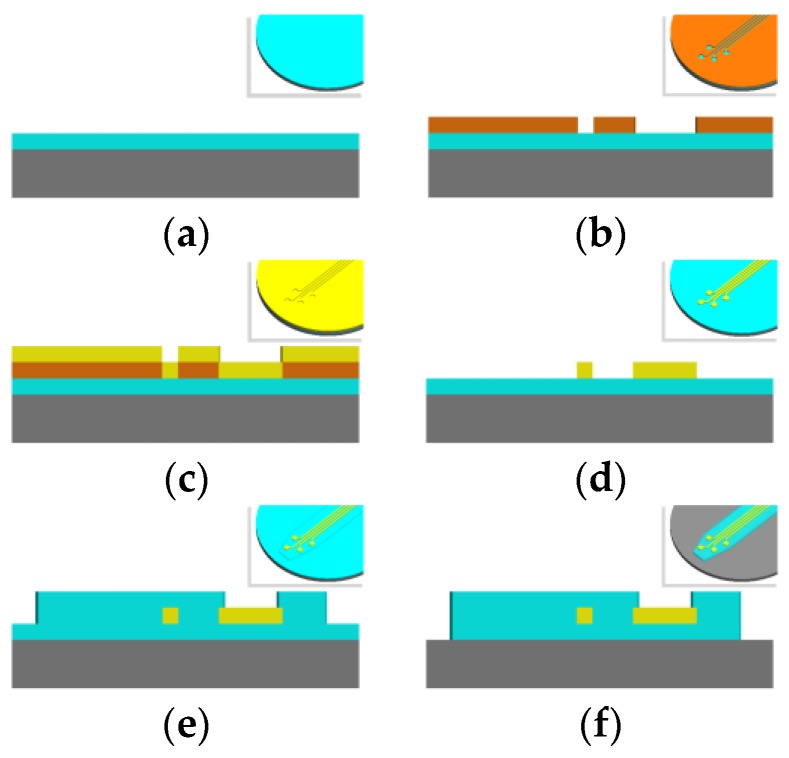
Fabrication process template in cross-section and isometric views (inset) in the following processing steps: (**a**) substrate polymer deposition; (**b**) sacrificial layer deposition and photolithography; (**c**) metal deposition; (**d**) sacrificial layer removal; (**e**) insulation polymer deposition and etching; and (**f**) outline etching and release.

Microfabrication of microelectromechanical system (MEMS) devices is accomplished layer-by-layer in processing steps that involve a combination of deposition, lithographic masking, etching, and cleaning [31]. Permutations of these processing techniques are used to create the basic structure of a flexible intracortical probe that is conserved almost universally across all micromachined designs. This structure consists of a thin film metal conductor sandwiched between two layers of polymer, which echo the planar nature of microfabrication processing (Figure 3). The conductive layer is selectively exposed at electrode sites which are used for electrophysiological recording or stimulation. The resulting thickness of a probe is dominated by the resolution of film (polymer and metal) deposition while the lateral features, such as the length and electrode size, are governed by the lithography and etching processes. This basic microfabrication template can be modified to create more novel cortical probe structures, some of which will be discussed in “Design Considerations” below.

A glass or silicon wafer is typically used as a carrier for fabrication and devices are removed from this carrier after devices are fully processed [32]. The type of additive or subtractive processes employed to create a device depend on how amenable the processing parameters are to the substrate and conductive material of choice. In polymer-metal-polymer devices, metal is deposited on top of a base layer of polymer via evaporation, sputtering, electron beam deposition, or other techniques. The metal can be patterned by means of lift-off or etching. Next, another layer of polymer is deposited on top of the entire wafer, insulating all metal traces inside. As a final step, the electrode sites and contact pads are accessed by reactive ion etching (RIE) or deep reactive ion etching (DRIE) of the polymer insulation that lies atop these sites, once again employing lithography to obtain the desired etching boundaries. This etch step may also fully cut the outline of the device for lift-off. Various cleaning steps are peppered throughout this fabrication scheme in order to clean the surface from contaminants prior to a deposition step or to roughen the device surface to enhance adhesion between structural layers.

### 2.2. Polymer Choices

Having established the potential advantages in immune acceptance of compliant probes over rigid ones, material choice of the compliant probe is an important first consideration for discussion. It is vital to choose a material that can be chronically present without causing chronic damage and inflammation at the site of injury. The USP Class VI and the ISO 10993 are two standards by which to measure the biocompatibility of a material [33,34]. However, the rigorous tests required to achieve these standards are rarely performed by the material manufacturer. Instead, the biocompatibility of a polymer must be investigated by the end-user prior to design or fabrication. Additionally, material properties are an important concern in establishing the types of processing that the material can undergo without being damaged or otherwise compromised. For example, it is essential to ensure that the processing steps do not exceed the temperature limits of the polymer. For an in-depth discussion of the physical properties relevant to microfabrication of polymers for neural implants including penetrating probes, the reader is directed to work by Kim et al. and Hassler et al. [35,36]. A summary of relevant polymer properties appears in Table 2 and a few prominent polymers, along with their properties of interest and disadvantages, are discussed in the text below.

Polyimide: Polyimide has found wide use in electronics as an insulation layer due to its low moisture penetration and high thermal and chemical stability and is increasingly becoming a more common material for biological applications [35]. The polymer can be purchased as a thin film or an adhesive tape, but for MEMS fabrication, it is usually used as a resin, which is spun into a thin layer and then cured. One variant of polyimide, called Durimide, made by Fujifilm, can be photopatterned. Polyimide resin does not have a USP or ISO rating, however fully cured polyimide tubes and sheets are advertised as such. Many groups have continued to evaluate the effects of polyimide in the brain [37,38], and one recent study confirmed that even the photosensitive formulation caused no toxicity after six months of implantation in a rabbit retina [39].

Parylene: Parylene is the trademark name of a group of poly(p-xylylene) polymers, the most notable of which is Parylene C, as it was the first Parylene type to receive USP Class VI and ISO 10993 compliance. A unique feature of Parylene is that it can be coated by chemical vapor deposition (CVD) in vacuum, which enables conformal coating. Parylene C exhibits chemical inertness and is pinhole-free. Because of these advantages, Parylene C is frequently used in Food and Drug Administration (FDA) approved implants.

SU-8: Though initially developed as a sacrificial, negative photoresist in the electronics industry, SU-8 has reemerged in biomedical research applications. SU-8 can easily be patterned to be thick, with high-aspect ratios, which make it amenable to use as a mold for other structures such as microfluidics or as a core material for neural probes [10]. SU-8 received a rating of less than “mildly reactive” according to ISO 10993 standards [47]. Even though there is no formulation of SU-8 that has been confirmed to be suitable for medical device use by the USP or ISO, studies suggest its low cytotoxicity and suitability for implantation [48]. We note that SU-8 is not supported for in vivo use by the manufacturer nor is it found in any FDA approved implant to the knowledge of the authors.

Polydimethysiloxane (PDMS): PDMS has been used widely in microfluidic and non-penetrating neural arrays applications, but has rarely been used for penetrating probes due to its high flexibility [49]. Instead, PDMS often plays the role of a mold material or structural material for a secondary structure such as microfluidics [50,51,52]. On occasion, PDMS may be used as a coating for freestanding metal [53]. PDMS has high viscoelasticity, high permeability to gases, a low dielectric constant, and a low Young’s modulus, which can be altered by changing the curing temperature [54]. Additionally, medical-grade PDMS that meets both USP Class VI and ISO 10993 requirements is available commercially. Photopatternable PDMS formulations have not been fully evaluated for biocompatibility. Of the many formulations of PDMS that exist, several are medical grade and commonly used in FDA approved medical devices and implants, including deep brain stimulators and ECoG arrays. PDMS is cured from a two part elastomer and curing agent mixture, which can be spin coated or casted on a substrate. However, some studies provide evidence indicating that the curing agent may be toxic. Therefore, users are advised to mix, cure, and sterilize their PDMS thoroughly before using it for a biological application [2,55].

Each of the polymers listed above has successfully been used as a substrate in flexible neural probes, but many groups are concentrating their research efforts on the investigation of the viability of newer polymers. Lee et al. successfully created a three-pronged probe from benzocyclobutene (BCB) using photolithography—spin coating, light exposure, and then development [42]. Though BCB seems promising in acute in vivo studies, long-term studies have yet to be reported [2]. Another new material used for neural probes is liquid crystal polymer (LCP), a semi-crystalline aromatic polyester, which one group fabricated using laser micromachining and thermal bonding [56]. Though in vivo biocompatibility of LCP was not demonstrated with this device, a non-functioning LCP-based retinal device was implanted by the same group for 2.5 years with no sign of damage or inflammation in the subject [57].

Some of the newer polymers under investigation have the unique ability to modulate their elasticity. The Zorman group has investigated the use of polynorbornene (PNB) as a possible material for flexible implants. The Young’s modulus of PNB decreases from 887 to 742 MPa after soaking in saline and this drop in stiffness can be useful for improved integration with brain tissue [58]. Earlier work by this group in cell culture studies indicates that it may be safe for use in acute biological applications [59]. PNB can be fabricated using standard photolithography. Inspired by the changing behavior of the sea cucumber skin, the Capadona group has developed a polymer nanocomposite—tunicate-derived whiskers dispersed in poly(vinyl acetate), called PVAc-TW—whose Young’s modulus changes from 3420 MPa when dry to 20 MPa when wet, as another potential substrate material. However, PVAc-TW is incompatible with typical MEMS fabrication methods, and as such must be used in conjunction with another substrate, such as Parylene [60]. Another adjustable polymer, off-stoichiometry thiol-ene-epoxy (OSTE+), has a glass transition temperature that is manipulated as its stoichiometry changes [61]. By tuning this property, OSTE+ is rigid at room temperature but flexible at body temperature; for one blend of OSTE+, its Young’s modulus changed from over 1 GPa to 30 MPa.

The polymers listed above vary in their chemical and material properties, ability to interface with biological tissue with minimal interference, and processing abilities. In the following section, we discuss the means by which these polymers are commonly deposited.

### 2.3. Methods of Polymer Deposition

The method used to deposit the base polymer layer of devices is dictated by the material chemistry. All materials mentioned here have been demonstrated to be biocompatible to different degrees [2] and are more flexible than traditional materials for penetrating cortical probes (e.g., silicon, stainless steel, or glass), but they vary widely in their ability to be processed and possess different process limits, which, if exceeded, may compromise the material itself.

Polymers used for additive fabrication of the base and insulation layers in penetrating brain are most commonly added by means of either spin coating or chemical vapor deposition. During spin coating, the thickness of the deposited material is controlled by the viscosity of the uncured polymer, the rotational velocity of the substrate, the flatness and properties of the surface onto which it is deposited, and resultant shrinkage during solvent curing [20]. Spin coating is the most common way to deposit polydimethylsiloxane (PDMS), polyimide, and SU-8 for neural probes [10,20,36,49]. Parylene C, on the other hand, is applied to surfaces via a process akin to chemical vapor deposition (CVD) [8,10,30,53,62,63,64,65] that results in conformal coating over the entire surface. In this process, thickness is controlled by the amount of initial dimer fed into the chamber, and its relationship with the magnitude the surface area to be coated.

Optionally, these polymers can be cast into a mold [36,49,66,67], which is one method of creating structures with high-aspect ratios [68]. Liquid prepolymer can be flowed into a pre-fabricated mold, and polymers that are traditionally deposited via chemical vapor deposition can be deposited on top of a mold directly. However, difficulties in molding arise from the small scale of MEMS fabrication, in which capillary forces dominate fluid, inertial, and viscous forces. Structures with smooth surfaces and sharp corners grow more difficult to resolve as the scale of the mold grows smaller. Additionally, molds for materials that are deposited by means of CVD are difficult to separate, and may require the use of adhesion-promoting or adhesion-demoting techniques to [67] help decouple the mold from the substrate. Molding is routinely used in the fabrication of non-penetrating arrays that lie on the surface of the target tissue, such as electrocorticography [69], spinal cord stimulation [70], and retinal prosthesis [71]. However, for neural probes, molding is most often relegated for coating devices with stiffeners or biologically-inspired chemicals, rather than for use in the fabrication of critical features.

Other deposition techniques include screen printing PDMS or extruding polyimide [72], but these have not been reported in the construction of penetrating neural implants.

Although polymers can be successfully deposited with the techniques mentioned above, many challenges with polymer MEMS structures can arise during subsequent fabrication steps, such as deposition of a conductive layer. Polymers are unable to tolerate high temperatures during processing, so the range of feasible metallization techniques is narrower than it is for silicon whose melting temperature is much higher. Even with metal successfully deposited, there could be issues of thermal stress caused between the large difference in thermal coefficients between typical metals and polymers and some polymers experience poor adhesion with metals. Strategies to improve polymer-metal adhesion include the use of more reactive metals, like titanium, as an adhesion layer, chemical adhesion promoters [73,74], mechanical interlocking mechanisms [75], annealing [76], and plasma treatment of polymer surfaces [77]. For a detailed analysis of adhesion strategies for SU-8, polyimide, and Parylene C see [35].

### 2.4. Choice of Conductive Layer: Deposition, Patterning, and Electrode Surface Modifications

There are also a variety of materials to choose from when it comes to determining what type of conductive layer to use for neural probes and how to best deposit these conductive layers onto the substrate of interest. Electrode sites should ideally lie within a 50–100 µm distance from surrounding neurons in order to acquire resolvable, single unit electrophysiological signals, and should not exceed a distance of 140 µm [78]. Since electrodes are in direct contact with fluid (the saline environment of the body) or tissue within the brain, the metal selected must be corrosion resistant; corrosion could result in a release of toxic substances into neighboring tissue. This criterion is met by noble metals and metals that form stable oxide layers [79], among which gold, platinum, iridium, and titanium exhibit high conductivity. Titanium alone can oxidize in the body, and so is used primarily as an adhesive layer [80,81], and electrodes made of pure iridium, which has a much higher melting temperature than either gold or platinum, are rare in part due to the difficulty of depositing this metal onto polymeric films [71,82]. Futhermore, iridium oxide may be preferred for its improved charge properties [83].

Thin film gold, platinum, titanium, and aluminum (a frequently used etch mask and sacrificial material [30,49,67,84]) layers are commonly deposited on polymer substrates via physical vapor deposition techniques including thermal evaporation, e-beam evaporation, and sputtering [31]. Other metal deposition techniques are available, but few can process substrates at temperatures low enough to safely accommodate polymers. Sputtering can be used to deposit metal alloys by controlling the flow rate of a secondary species such as oxygen or nitrogen along with the evaporated metal. For neural applications, iridium oxide and titanium nitride are common electrode material choices, though iridium oxide possesses better properties for both recording and stimulation [85,86,87]. Iridium oxide may also be produced by electrochemically activating pure iridium [86].

Patterning of electrodes and electrode traces can be accomplished with a variety of methods, but the ones most simple and frequently used are using etching of the conductive layer itself or lift-off photolithography—either by standard ultra violet (UV) photolithography, or e-beam lithography in which an electron beam is used to expose the sacrificial layer [32]. E-beam lithography offers high resolution, and with more rigid substrates such as silicon, nanoscale feature resolution is possible. With softer polymers like photoresist, however, there exists a “proximity effect” where electrons from the beam may scatter into adjacent areas, creating nearby sites of unwanted exposure. Experiments to find the proper dosage is critical to limit these spreading effects [79].

Another method of patterning a metal layer is by transfer printing, especially in cases where the conducting material cannot be directly deposited onto the polymer. In this process, a pattern is deposited onto a temporary material that serves to stamp the metal layer onto the substrate of interest [79]. Provided that the adhesion between the pattern and the substrate is stronger than between the pattern and the stamp, the pattern will be transferred to the substrate in a process that can be temperature-independent. In a recent example of transfer printing as applied to MEMS devices and flexible materials, Fan et al. applied a boron-doped polycrystalline diamond (BDD), whose growth temperature is 500–900 °C, to Parylene C, a substrate with a glass transition temperature of 90 °C (Table 2) [88]. In another example, David-Pur et al. grew multi-walled carbon nanotubes (MWCNT) at 900 °C, and then successfully transferred a pattern of MWCNT using medical tape, Parylene C, polyimide, and PDMS [89].

Another alternative microfabrication technique, laser micromachining, has been utilized with both polymers [56,90] and metals [2,53] in place of lithography. One drawback to laser machining is the fact that it cannot be integrated with a batch process, causing longer process times than other deposition techniques. Additionally, if any heating occurs during laser machining, the cut depth may vary across devices.

Once a conductive layer is added to a neural probe by any of the deposition processes mentioned above (etching, photolithography, transfer printing, or laser micromachining), its quality determines whether further processing is necessary to enable the conductive layer to meet the needs of the probe. The surface quality of electrodes for both recording and stimulation is of utmost importance, especially for the smaller, more densely packed electrodes that are necessary for ideal discrimination of individual neurons. Merely decreasing the size of electrodes, however, leads to unfavorably high electrode impedances for recording electrodes. Stimulation electrodes have their own, attendant, challenges as they require higher charge densities than recording electrodes [91]. Electroplating offers a method through which the effective surface area of an electrode can be increased, to decrease its impedance [50,71,92], or by which a new material can be deposited on top of an existing electrode, to change its injection capacity and impedance [93].

Electrodeposition of a material that is different from the electrode is not limited to metals, and a growing area of research is the exploration of the use of conducting polymers (CP) to improve electrode characteristics. Of these unique materials, the most established are polypyrrole (PPy) and poly(3,4-ethylenedioxythiophene) (PEDOT) [1]. Multiple studies have demonstrated that CP coatings added to gold electrodes can lower the impedance and raise the charge density of the bare electrode [94,95,96,97]. George et al. were able to fabricate an implant entirely out of PPy which acted as a single large electrode [98]. More recently, Castagnola et al. investigated electrodes on a Parylene C neural probe and confirmed that gold electrodes modified with a PEDOT coating had improved electrical characteristics [99]. For further discussion of conducting polymers, particularly PPy and PEDOT for neural probe applications, see work by Guimard et al. [100].

The deposition and modification processes for conductive surfaces described above have been creatively applied by some researchers in novel ways to achieve other improvements for flexible neural probes. For example, Fomani et al., in pursuit of a thick metal layer with a high aspect ratio, used electroplating as a way to fabricate thick gold layer to mechanically reinforcement their flexible neural probes, in order to enable probe penetration without buckling [98,101]. A novel approach to electrode positioning has been demonstrated by Metz et al. Electrodes are typically recessed from the surface of the neural probe as the top insulative substrate of the probe is selectively removed above electrode sites. This group was able to fabricate electrodes that lay flush with the probe surface to bring electrodes closer to surrounding neurons by first patterning the substrate layer of their probe prior to metal deposition [102].

## 3. Design Considerations for Fabricating Flexible, Neural Probes

The many different substrates and fabrication techniques that were honed for the creation of flexible, polymer-based neural probes, have provided a rich tool set to bolster creative approaches to probe geometry and design in the hopes of enabling longer probe lifetimes. Traditional probes take the shape of planar needles, with sharp probe tips and circular surface electrodes sitting along the probe midline. Some novel designs include the addition of lateral anchors to help keep the probe in position over time, inventive techniques for minimizing the cross-sectional area of the probe, placement of electrodes away from the damaged insertion tract, open-architecture constructs, and original methods to effectively “untether” the flexible probe from its attachment to the cranium.

### 3.1. Using Anchors to Attenuate Micromotion of Flexible Probes

**Figure 4:**
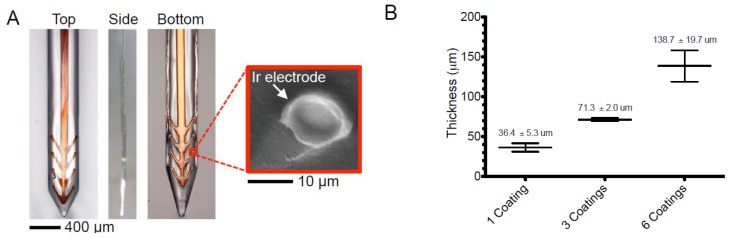
(**A**) Example of flexible polyimide probe coated in silk for temporary stiffening during insertion. Anchor-like protrusions from probe designed with intent to minimize probe micromotion in brain. Opening angle represented by angular spread of probe tip: 60° and 30° opening angles in top and bottom views, respectively; (**B**) Graph describing relationship between number of silk coatings and total device thickness. Reproduced with permission from [90].

Some groups have explored the use of barbed structures that function as local anchors between the implant and tissue to attenuate if not eliminate micromotion between the two. Tissue deformation is thought to displace the electrode positioning relative to surrounding neurons, and movement of the probe, especially one with a sharp rigid edges, can cause physical damage to surrounding tissue. The first report entailed incorporating microfabricated flanking anchors that protruded backward from either side of an SU-8 based penetrating probe at an angle of 60° from the horizontal [103]. Electrode recording sites were patterned onto each anchor. However, the effectiveness of these anchors were not evaluated in that study. The same group repeated a similar design, with more electrodes on a stepped, barb geometry, to achieve more durable anchorage and attenuate tissue reactions arising from the large separation between the probe shank and its electrode sites [92]. Similarly, fish-bone shaped polyimide probes with each branch serving as a separate anchor point were explored [104] and a sinusoidal shaped Parylene probe with a different anchor design—a polyimide ball at the tip of the electrode—was examined as well [30]. Histological and lifetime testing of these and other [53] anchored designs compared to unanchored probe counterparts are lacking and thus their specific advantages remain unclear. See Figure 4 for an example of barbed structures built in to a polyimide probe. It is important to note that the use of mechanical anchors built into the structure of the probes may complicate probe retraction in future human trials that necessitate surgical revisions. Forcibly retracting an anchored probe can easily damage tissue around the implant. As such, the potential benefits of attenuating probe micromotion through the use of anchor or barbs must be weighed against the prospective damage these structures can cause during probe removal.

### 3.2. Minimizing Cross-Sectional Footprint of Probes to Decrease Immune Response

It is presumed that implants with smaller footprints will displace less tissue and produce a surgical tract that causes less damage to surrounding vasculature and neurons. Experimental evidence supports the idea that minimizing cross-sectional areas of probes can alleviate the magnitude of the resultant immune response. One study of tissue response to polymeric fibers of varying diameters found reduced macrophage density on probe fibers with diameters of <6 µm [105]; this report supports the results of a prior study which indicated that smaller diameter fibers in the range of 2–12 µm resulted in minimal macrophage spreading on fibers in cell culture studies [106]. While these studies imply that implants beneath a certain size threshold may be able to hide undetected by immune cells, in vivo studies evaluating this hypothesis reported conflicting results.

Studies on implanted silicon probes by Szarowski et al. indicate that implant size determines only acute tissue damage, but that the chronic “kill zone”, which determines the distance between electrode to neuron, is independent of implant size and is instead due to factors inherent in the device-tissue interaction [107]. This suggests that minimizing the size of implant would only offer a temporary, acute benefit, but that over time (within four weeks) this advantage wanes. It is possible that the extent of chronic damage is so large in rigid probe insertions that any benefits due to smaller sizes of probes are masked. Similar histological studies were performed on compliant probes made of SU-8 encapsulated in Parylene with lateral lattice like structures of various thicknesses (4, 10, 30, and 100 µm). Though a 1/3 decrease in non-neuronal density and a 1/3 increase in neuronal density surrounding the lattice offshoots as compared to the bare shank were shown, this study revealed no statistical differences in immune reactions against lateral platforms of varying widths [10]. 50 µm diameter stainless steel implants were surrounded by significantly lower populations of astrocytes and microglia and higher populations of neurons as compared to 200 µm diameter controls at 6 and 12 weeks post implantation in rats [108]. Another study of stainless steel microwires coated with poly-glycolic acid (PGA) to aid in insertion found that probes with a 12 µm diameter experienced a significant reduction in GFAP expression at four weeks post implantation into rats compared to 25 µm counterparts [109]. Further studies are needed to determine whether or not smaller probe sizes increase the population of healthy neurons surrounding the implant.

### 3.3. Placement of Active Sites Away from Probe Body, or Deeper Along Body to Limit Tissue Disruption

Immunohistochemical analyses of the immune reaction against single penetrating probe implants (at four weeks in rat cerebral cortex) indicate that the tissue reaction is mostly confined to a 25 µm radius around the SU-8 and Parylene probes [10]. Additionally, minimal immune response was seen near electrodes placed on the lateral edge of the distant, Parylene lattice structures of various thicknesses, while traditional placement of planar electrodes exposed through the surface of the probe was shown to cause more severe scarring [10]. Collectively, these results indicate that placement of electrodes away from the probe shank may be advantageous. Several groups have studied this in acute experiments [92,103,104], but chronic neural recording evidence is needed to evaluate the long-term impact of this technique. In a study of silicon probes inserted into rabbit cerebral cortex, severe tissue reactions formed at the sharp tips of the probes and at the sharpened edges along the length of the probe, causing the authors to suggest placing electrode sites at a distance from these areas [110].

In light of these findings that suggest placing the electrodes away from the body of the probe, it is interesting to note that finite element modeling of tissue strain indicated that induced strain was highest at the surface of the implant, which may indicate a potential advantage in probes targeting deeper brain structures with electrodes located farther away from the brain surface [28]. This idea is also supported by a study that reported non-uniform microglial coating of penetrating probes, with denser microglia attached to superficial areas of the probe. This same study discovered the presence of vimentin-staining, non-central nervous system (CNS) cells surrounding the implant, leading to the conclusion that cells of non-CNS origin, perhaps meningeal cells, are dragged in during probe penetration [111]. Another study, evaluating the histological effect of insertion parameters on silicon probes, also noted more neuronal death at superficial shank locations. This was hypothesized to be due to damage caused during tissue dimpling before penetration during implantation, bone or other material debris infiltrating from the surgical site, or the fact that tethered probes may damage surrounding tissue more severely at locations close to the point of tethering [112]. These discoveries raise the possibility that deeper electrodes are more protected against elements that disrupt electrical recordings over time.

### 3.4. Open Architecture Design

While it has been posited that open architecture probe designs can help improve integration between implant and tissue, aid in diffusion of nutritional molecules between tissue on opposite sides of the implant, and help re-establish communication between populations of cells that were disrupted during probe insertion [10], there is insufficient in vivo data to support or contradict these claims. In the study examining Parylene lattice structures protruding from a central shank, dense encapsulating cells were yet present within the open architecture regions [10]. A syringe-injectable SU-8 mesh with platinum recording sites located at each junction of the lattice was associated with promising histological results. After 5 weeks of implantation in rodents, NeuN (staining for neurons) was found to tightly associate with the mesh, and GFAP levels indicated that astrocyte levels were similar to that in control brain tissue. The authors of this work attribute these positive cellular interactions to the small feature size of the mesh (ribbons of the mesh were 5–20 µm in width, and <0.4 µm in thickness) as well as the flexibility of their substrate. However, it is quite possible that some benefit was derived from the open architecture design of the mesh, which enables tissue to chemically communicate around the implant [113].

### 3.5. Untethered Probes

When probes are anchored to the cranium, any movement of the brain relative to the skull has the potential to dislodge the placement of the probe. Respiration has been shown to cause 80–130 µN of force acting against the probe by surrounding brain tissue, while vascular forces corresponding to a frequency of 5–6 Hz caused impacts of 14–25 µN on stainless steel microwires implants [114]. This was shown to correspond to probes moving 2–25 µm during respiration and 1–3 µm during cardiac pumping [115]. Micromotion of the brain against tethered implants has been posited to increase the amount of microdamage around the implant, particularly in probes with sharp tips and edges. In a study of tethered and untethered silicon microelectrodes implanted into rat cortex, tethered microelectrodes were shown to elicit upregulation of ED1 and GFAP suggesting increased density of microglia and astrocytes around tethered implants [116]. A similar study with stainless steel implants in rats showed the same results at 6 and 12 weeks post rat implantation [108].

Untethered probes can potentially eliminate this source of damage. However, traces from probes that are not wireless must pass through the animal’s cranium and connect to subsequent wiring for recording and/or stimulation. Creative solutions that mimic an untethered state, while passing through the animal skull are necessary before wireless solutions become available. The earliest group to address this, to our knowledge, created an “s” shaped cable leading up to the probes, in order to lessen the transfer of movement forces between the cranium and probe [20]. Another group explored the design of a probe with a sinusoidally shaped shank in an attempt to mechanically decouple the recording site from the point at which the shaft is tethered. This study included two years of in vivo data that suggest less astrocytic infiltration around the sinusoidal probe as compared to a traditional microwire [30]. Probe untethering can be applied to the mechanical decoupling of each trace from its neighbor as well as between the recording end of a probe and its attachment to the cranium. One study using such an approach created milled gold traces coated in Parylene, held in place by a gelatin/polyethylene glycol (PEG) matrix. Once inserted into the brain, the matrix dissolved, enabling free movement of each separately insulated electrode trace in all three directions [53]. However, compressive forces within the brain may expunge the untethered probe with time [112]. This concern has yet to be evaluated in current literature.

## 4. Insertion Requirements

### 4.1. Determining Force of Penetration

In order to achieve successful surgical placement of neural probes into brain tissue, probes must be mechanically robust enough to penetrate brain tissue. The penetration force must exceed the buckling force threshold of the neural probe. Flexible neural probes often require mechanical augmentation to temporarily increase probe stiffness above that required to achieve insertion without buckling. For polymer probes, there are two dominant approaches to mechanical augmentation including increasing probe dimensions or the use of an insertion shuttle. Although increasing the probe dimensions will increase the buckling force threshold, a permanent increase in stiffness would obviate, in part, the advantages gained by using a softer polymer substrate. Therefore, the most common approach is to temporarily increase probe dimensions and stiffness using soluble coatings or insertion shuttles.

First, tissue properties relevant to surgical implantation are discussed followed by probe design considerations and the selection of surgical insertion speed as well as the manner in which these factors have been shown to affect penetration force.

#### 4.1.1. Tissue Properties

Properties inherent to target tissue, such as elastic modulus, are main players in the determination of penetration force. Tissue properties depend on the species of the subject animal, the age of the animal, stiffness characteristics of the brain target, and the presence of the dural covering of the brain surface. All four of these tissue-specific variables are critical in determining the penetration force of neural probes for the particular application of interest.

Animal species can differ in brain structure, organization, and tissue properties. For example, though rat, porcine, and mouse brain tissue are similar in elastic moduli, the former two moduli are often lower than that of mouse brain tissue, indicating a species-specific effect [11]. Tissues of higher elastic moduli require a larger penetration force. See Table 1 for a comparison of elastic moduli over analogous locations in different species. Ranges in elastic moduli are due to differences between measurements from indentation and tensile tests, whether or not the sample is constrained during testing, age of the species measured, and the fact that the viscoelastic properties of brain tissue are time and depth dependent [12,13,117]. It is important to make note of the Young’s modulus of a particular animal subject’s target location prior to designing probes that can withstand buckling caused by forces applied to the probe during penetration.

Not only does modulus differ by species, but it is highly dependent on animal age as well. Elkin et al. showed that the mechanical stiffness of rat brains increases by over 100% with age, a phenomenon attributed to a decrease in water content and increase in protein content over time [13]. In another study, cerebral arterioles of aged rats (24–27 months old) were found to have higher collagen to elastin ratios than their younger, adult (9–12 months old) counterparts [118], which also contributes to stiffer tissue [119]. Another study showed a two times decrease in the elastic modulus of rat brain as a rat developed, and attributed these changes to increased myelination during development which, with its high lipid content, actually makes the brain softer. This study compared rats that were 2–3 weeks old to older rats, of ages 6–12 weeks [119]. Taken together, these two studies seem to indicate that the stiffness of a rat’s brain can change during different developmental time periods with reduced values in the early stages of a rat’s life and increased stiffness as a rat ages through adulthood.

Target location intuitively can also affect penetration forces. After the neural probe penetrates through the meninges, it travels through tissue containing neurons (with their attendant cell membranes and their processes ensheathed in myelin), glial cells, and small vasculature before reaching its target. Microtubules (260 Å diameter) and neurofilaments (100 Å diameter) are fibrous structures that impede the path of travel of penetrating probes—any implant larger than a few tens of angstroms can catch on these structures and either tear, cut, or stretch the impeding tissue [110]. Once the probe bypasses these obstructions as it passes to its target site, it must then contend with the target tissue, which can exhibit different stiffness values.

Different anatomical locations in the brain have different underlying structures, including cell types, density of cell populations, and variations in vasculature, all of which contribute to the resulting stiffness value. Atomic force microscopy indentation tests performed by Elkin indicate that juvenile rat cortex is stiffer than the hippocampus, but that moduli measurements, on average, become more similar as rat tissue ages [13]. Another study evaluated the forces required to penetrate mouse cortex compared to mouse olfactory bulb and found the magnitude of penetration forces to be similar, but the subsequent frictional forces experienced as the probe is lowered further into brain tissue differed significantly due to the presence of the underlying fibrous tracts of the corpus callosum lying beneath the olfactory bulb [11]. The dura, the outermost meningeal covering of the brain made out of dense connective tissue, has a higher Young’s modulus than cortex, the cerebellum, or the hippocampus (Table 1). Sharp et al. compared penetration forces required with and without the presence of the overlying dural meningeal membrane and found that forces required to insert with the dura and pia layers present were orders of magnitude larger than those required when those two superficial meningeal layers were removed. Many groups, acknowledging this challenge, prefer to remove the dense, collagen matrix of the dura prior to probe implantation to aid in ease of insertion. The dura can be removed with sharp forceps, and the remaining arachnoid and vascularized pia layers of the meninges are removed through gentle swabbing of the brain surface [110].

#### 4.1.2. Geometrical and Probe Surface Considerations

In addition to properties inherent to target tissue, geometrical considerations of the implant also contribute to the determination of penetration force. Considerations of interest include probe (and, of course, insertion aid) cross-sectional dimensions, the sharpness of the neural probe tip (opening angle of the probe), as well as the cleanliness of probe tips and the roughness of the surface. Each of these geometrical considerations plays a role in determining a penetration force value that probes must be able to withstand during insertion surgeries.

Cross-sectional dimensions of the probe shank have been shown to affect the force required to penetrate brain in experiments that studied blunt-tipped microwires in vivo. Tungsten probes with a diameter of 50 µm, were shown to have a rat pial penetration force of 0.85 ± 0.33 mN, compared to a penetration force of 1.15 ± 0.51 mN for 150 µm diameter tungsten probes [120]. Additionally, probes of stainless-steel wire with larger cross-sectional areas (100 µm diameter) required two to four times higher force to penetrate tissue than their narrower, 200 µm diameter counterparts [11]. These results are intuitive as larger diameter probes displace larger tissue volumes than thinner probes do during insertion. According to another study, minimizing the width of a silicon probe was one of the most influential factors in reducing the end forces experienced by the probe after it was placed in its target location [121]. The only study of insertion mechanics, to the knowledge of these authors, that directly evaluates flexible probes, found that polyimide probes had penetration forces that were two orders of magnitude less than their silicon, glass, and tungsten microwire counterparts. The thickness of the polyimide probe in this study was 10 µm compared to the ~100 µm thicknesses of the other probes, which could be a factor in its smaller penetration force [122].

**Figure 5:**
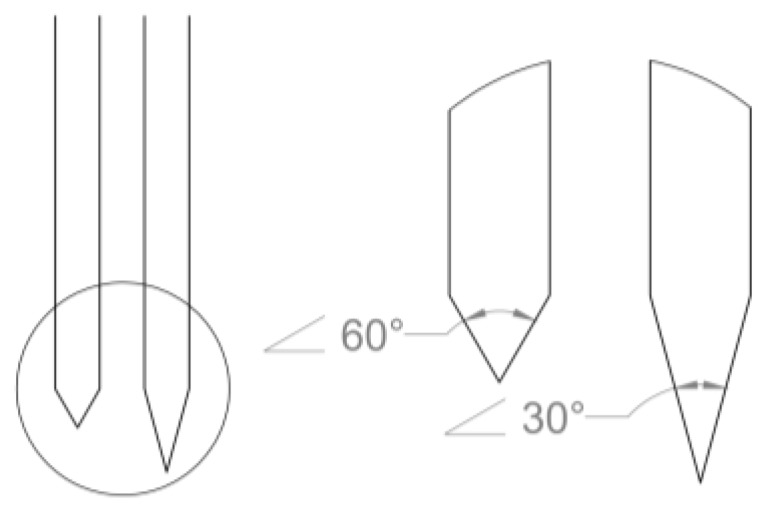
The opening angle of the probe is defined as the acute angle formed at the tip of the probe from edge to edge. Inset illustrates a probe with an opening angle of 60° and 30°.

Not only is the cross-sectional size of the probe important in determining penetration force, but the tip shape of the probe is as well. The larger the opening angle of the probe, the higher the penetration force [120]. The opening angle is defined as the acute angle formed at the tip of the probe from edge to edge (Figure 5). This may be due to the fact that sharper probe tips sever less tissue during penetration and allow for displaced tissue to spread more easily over the remainder of the probe. Probes with opening angles of <20° were shown to easily penetrate the dura without dimpling the surface, whereas probes with opening angles >40°–50° made penetration of the dura difficult [110]. Tip angle, in another study of silicon probes, was shown to be one of the greatest determiners of insertion force. Making the tip angle of the probe smaller or decreasing insertion speeds, exponentially reduced probe insertion forces necessary to penetrate pia [121].

The cleanliness of the probe has been shown to impact penetration forces. Cleaning treatments with either Piranha or silane solutions to make the probe surface more hydrophilic lowered penetration forces by 0.5–1.0 mN and resulted in less tissue dimpling before penetration [120]. In another study, coating silicon probes that were etched by a Bosch process with Parylene helped smooth the sidewalls, and reduce friction between the implant and brain tissue during insertions to deeper target sites [121]. These geometrical and surface considerations may serve as guiding factors by methods for which to decrease forces experienced by the probe during in vivo implantations.

#### 4.1.3. Variations in Insertion Speed

The precise insertion speed to surgically implant a probe is the subject of debate and conflicting results in the research literature further confound the selection process. While rapid insertion can compensate for poor tip design by allowing for penetration in circumstances where geometrical considerations would make penetration prohibitive, slower insertion speeds may be advantageous in allowing time for surrounding tissue to accommodate around the probe, thereby attenuating compressive and tensile forces of surrounding brain tissue [110]. Traditionally, insertion speeds range from slower insertion speeds around 10 µm/s to as high as 1 mm/s. Penetration force for sharp-tipped silicon probes was shown to decrease exponentially as insertion speed was reduced within the range from 100 to 10 µm/s [121]. While lower penetration forces were reached for stainless steel wires inserted at speeds of 10 µm/s compared to 1 mm/s [11], which was hypothesized to be due to the viscoelastic nature of brain tissue which causes an increase in brain stiffness as insertion speeds increase, others reported no significant difference between penetration forces for Parylene coated silicon probes implanted at different insertion speeds in this range [112]. Other studies seem to indicate that slower insertion speeds cause greater degrees of biological damage. In one study, insertion speeds of 125 µm/s, on the slower end of the range of insertion speeds, actually caused increased vascular damage, independent of tip geometry, as compared to faster insertion speeds of 2000 µm/s in an ex vivo study [123]. This is supported by another study, in which specialized electrodes for extracellular pH recordings noted longer periods of acidosis, a witness for tissue homeostasis and the presence of blood along the insertion tract, for slower (50 µm/s) insertions as compared to faster insertions at 0.5 or 1 mm/s [124]. In another study, exploring the same range of insertion speeds, using an electrochemical force sensor to evaluate insertion mechanics, researchers found that although increasing insertion speeds lowered normal forces experienced by the probe, they increased the shear forces caused by friction between tissue and probe faces [125]. This may explain why slower insertion speeds are reported to decrease penetration forces, but counterintuitively increase vascular damage and cause more disruption to brain tissue homeostasis. Future studies, accounting for the viscoelastic properties of brain and both axial and shear forces acting on the probe, are necessary to explore this seeming contradiction. As arrays of neural probes become packed more densely together, limits of tissue displacement within the immovable cranium may be reached, no matter the speed of insertion.

#### 4.1.4. Current Consensus Regarding Penetration Force

Variations in each of the three variables listed above complicate the determination of a “universal” metric of penetration force for probes entering brain tissue. In one study that used silicon probes to penetrate rat cerebral cortex, penetration forces were found to range from 0.48 to 2.42 mN (for the initial insertion into pia), for arrays of varying numbers of shanks, with an average force of 1.45 ± 1.04 mN. The high end of this range of penetration forces corresponds to arrays of probes, and calculated force per probe measurements. If we narrow our focus to measurements taken for individual probes, the range changes to 0.48–1.15 mN with an average force of 0.775 mN [120]. Sharp et al. reported similar results for insertion of silicon probes into cortex and the olfactory bulb of rats, noting that penetration forces into cortex range from 0.54 to 2.48 mN, with an average of 1.25 mN, for individual stainless steel, cylindrical probes of varying diameter and tip shapes, inserted at various speeds [11]. The small discrepancy in penetration force ranges between Jensen and Sharp are to be expected since Sharp explored the insertion of totally blunt probe tips, whereas Jensen only examined probes with small opening angles (4° and 10°). Wester et al. determined the range of penetration forces into rat cortex to be between 0.5 and 1 mN accordingly [62]. Based on these data, an individual, tapered probe should be able to withstand 1.5 mN of force in order to penetrate rat cortex.

### 4.2. Buckling Force Threshold Calculations

Once penetration force has been established, it can be used as a design goal for buckling force threshold. By modeling penetrating neural probes as beams, clamped at one end and pinned at the other, many groups make use of the following calculation to estimate of the buckling force threshold of flexible, polymer probes. Probes are modeled as fixed at the base, since they are often reversibly adhered to the insertion platform and back-end electronics. They are considered to be pinned at the tip, since at the moment the tip contacts the brain surface, it is hinged but free to rotate.

The critical load that induces buckling in single beam (*P*_cr_) can be calculated theoretically using Euler’s formula [20]:

*P*_cr_ = (π*^2^Ewt*^3^)/(5.88*L*^2^) (1)

where *E* is the elastic modulus, *L* is the unsupported beam length, *w* is the width of the beam, and *t* is the beam thickness. This model makes the assumptions that the probe is a beam of a certain length, with a constant cross sectional area, that the neural probe is fixed at the top of its shaft during insertion, and that the tip of the probe cannot move in the *z*-direction [20]. Accordingly, one group has re-designed the geometry of their probes, particularly adjusting the parameters of probe length and cross section, to fulfill the minimum probe dimensions necessary to prevent probe buckling during insertion into brain tissue. Wester et al. made use of these buckling force threshold calculations to design flexible Parylene neural probes of 1 and 2.5 mm lengths (width of 100 µm, thickness of 25 µm) with buckling force thresholds greater than 1 mN that successfully penetrated rat cerebral cortex without any insertion aids [62].

## 5. Techniques for Temporarily Stiffening Probes During Insertion

**Figure 6:**
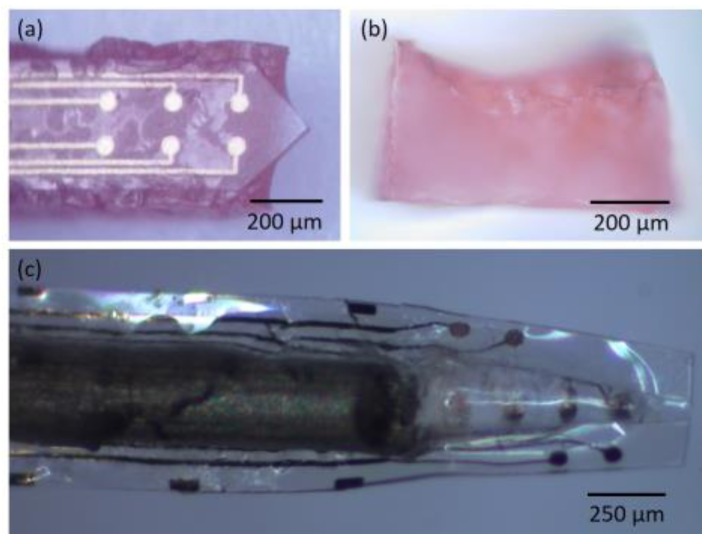
Example of a coating and insertion shuttle solution. Flexible Parylene probe coated in silk for temporary stiffening during insertion: top view (**a**), and cross-sectional view (**b**). Bare probe is 225 µm wide and 20 µm thick, with silk coating dimensions of probe increase to 350 µm wide and 236 µm thick, an 18-fold increase in cross-sectional area. Reproduced with permission from [126]. (**c**) Parylene probe with pocket fed with a 250-µm diameter tungsten push rod serving as a temporary insertion aid, removed after probe implantation. Bare Parylene probe is 450 µm wide and 5 µm thick. Insertion shuttle results in a 70-fold increase in cross-sectional area. Reproduced with permission from Brian J. Kim.

In circumstances where the desired fabricated dimensions of the flexible neural probe do not allow for a buckling force threshold that exceeds the penetration force, insertion aids are necessary for probe implantation. Many groups use either temporary coatings or temporary structural shuttles to boost the buckling force threshold of the probe during insertion. Each coating technique comes with attendant advantages and disadvantages. Both coatings and insertion shuttles increase the acute damage during insertion, as explained in Figure 6.

### 5.1. Coatings

Common coatings used to stiffen flexible neural probes during insertion include silk [90,104,126], polyethylene glycol (PEG) [19,126,127], tyrosine-derived polymers [128,129], carboxy-methyl-cellulose (CMC) [66], gelatin [53,130], saccharose [36], table sugar [131], and maltose [132] (Table 3). Biocompatibility of these substances is of utmost importance, as any exacerbation of the immune response to the coated probe by surrounding tissue detracts from the quality and lifetime of subsequent neural recordings. Coatings are biocompatible if they do not elicit an immune response from surrounding tissue, inherent to which is the assumption that byproducts of the coating can be effectively cleared from the surrounding brain tissue. No matter which application technique is used, coatings have the inherent disadvantage of increasing the cross-section of flexible probes, thereby undesirably enlarging the acute injury of penetrating probes. Coatings differ based on methods of coating and the time scale of dissolution of the coating. Coatings can chemically engineered together to adjust dissolution rates or achieve coating-specific benefits simultaneously [133]. According to Lewitus, successful coating solutions must be strong enough to allow for probe penetration, coating dissolution times must be quick enough to avoid a chronic immune response, and coatings byproducts must be safe [129]. Coatings should be uniform and smooth so as not to cause additional tissue damage during insertion [128]. Coating expansion due to moisture absorption from brain tissue post-implantation may sometimes lead to secondary damage [134]. See Table 3 for commonly used coating solutions in literature as well as their degradation pathways in vivo. It is important to be aware of tissue limitations in coating degradation and removal. One study of the use of maltose coatings for flexible probe insertions noticed that when coatings grew thicker than 180 µm in diameter, dissolution time in vivo increased dramatically, exceeding the body’s ability to transport away the sugar [132]. Another study demonstrated that CMC coatings may macroscopically diffuse from the penetration tract to the brain surface, as indicated by enlarged wound diameters in shallower parts of the cortex [134].

#### 5.1.1. Coating Materials

Silk: By increasing the β-sheet content of silk films through increased water annealing times, silk can be manipulated to have a tunable scale of dissolution (which ranges from minutes to days [138,139] and can therefore be directly tailored to the duration of implantation surgery in which temporary stiffening is required for probe placement [140,141]. Silk fibroin is degraded by proteases found in the body [142] and its byproducts have low antigenicity and non-inflammatory characteristics [135,142]. Additionally, silk fibroin can be easily sterilized as it is not damaged by the high-temperatures reached during autoclaving, and it can also be sterilized through treatment with ethylene oxide or ethanol [139,142].

Polyethylene Glycol (PEG): PEG is nontoxic, nonantigenic, and nonimmonogenic. In addition, it has been proven to reduce protein adsorption onto surfaces [143]. PEG is solid at room temperatures, liquid at temperatures greater than 50 °C, and water soluble, but the fate of its byproducts in vivo is unclear [129]. Though many have successfully adapted PEG as a coating for soft neural probes [8], others claim that it lacks the rigidity necessary to successfully serve as an insertion aide [128]. Ethylene oxide treatment of PEG may cause cross-linking of the PEG, and should therefore be used with caution as a sterilization method [133].

Tyrosine-derived: Tyrosine-based derivatives elicit only the mildest tissue response without inflammation [136] and dissolve into naturally occurring metabolites, tyrosine, and PEG within minutes to hours [128,129]. Addition of PEG and dityrosine further enhances control over degradation/dissolution rate. Incorporation of additional PEG into the tyrosine polymer back-bone causes the polymer to become more hydrophilic and accelerates degradation of the polymer [128]. Additionally, these derivatives maintain their properties after ethylene oxide sterilization [133].

Carboxy-methylcellulose (CMC): CMC is a naturally occurring, water soluble polysaccharide that is non-toxic [134]. CMC can be spin cast to mold it to a desired shape. CMC absorbs moisture directly upon contact with water, and swells into a gel at a rate controlled by the molecular weight of the CMC. This swelling may cause acute stresses around the implant and the body may respond by attempting to transport remaining CMC to the surface of the brain [66,134]. CMC clearance must be further evaluated for the determination of its potential success as a coating material.

Sugars: Sugars, such as poly(dimethylacrylamide) (PDMAA), glucose, sucrose, and maltose, begin to dissolve immediately (on the order of seconds) upon contact with water. Though one group has used a thin layer of mineral oil around the sugar coating to prolong the presence of the sugar coating [131], sugar coatings may not be suitable for longer duration implantation surgeries.

Other temporary coating materials: Poly(lactic-co-glycolic acid) (PLGA) has been used in past coating studies. However, its degradation rate is 3–4 weeks, which is too long to prevent damage to surrounding tissue due to a chronic immune response [129].

#### 5.1.2. Coating Methods

Coatings can be applied to neural probes either by dip coating or through the use of micro-molds. Micro-molds can confine the coating to area portion of the probe.

Dip-coating: Dip coating allows for uniform probe coverage and temporary stiffening of flexible neural probes. Inherent disadvantages to applying coatings to probes by means of dip-coating include difficulties in achieving the desired coating thickness (which depends on the concentration of the coating solution as well as the withdrawal speed from the solution), the blunting of neural probes tips (decreasing the effective opening angle of probes, causing penetration forces to be larger), as well as undesirable coating of electrode sites (which makes recording signals during implantation as a placement marker impossible if the coating does not dissolve quickly). One group presented a drawing lithography coating approach in which an increased temperature and speed of withdrawal at the tip of the probe preserved the sharp tip [132].

Micro-molding: The use of molds allows selective coating of neural probes focused on targeted areas (preventing exposed electrodes from being coated, for example), and does not blunt probe tips [90]. However, molds may be difficult to fill by hand and may require centrifugation to assist in the elimination of bubbles from the coating material, and stress-induced curling and shrinkage of the coated probe post-removal from the mold may interfere with the ability of the probe to penetrate properly during implantation [66,134]. Molds are traditionally made out of casted polydimethylsiloxane (PDMS) replicated from etched Si [134], SU-8 [126,128], or another appropriate negative mold. The PDMS mold may also be shaped via laser cutting or soft lithography [90]. Some groups use polyvinyl siloxane (PVS) [66] or polymethylacrylate (PMMA) [53] instead of PDMS.

### 5.2. Structural Shuttle

Shuttles are temporary structural supports that are another common way to achieve placement of soft polymer probes into brain tissue. Shuttles are attached to the back-side of a flexible probe, and for one-sided planar probes, pose no risk of covering up electrode sites during implantation.

Considerations of shuttle design dictate minimizing the shuttle cross-section, to minimize surgical trauma, choosing shuttle materials that are biocompatible and do not exacerbate the foreign body response, and fabricating shuttles out of materials that are easy to manipulate. However, if the flexible probe overhangs its stiff, shuttle backing, it is likely for the probe to get caught in superficial tissue during insertion. Therefore, shuttle bodies are made larger than the flexible probe to eliminate this issue. This need naturally conflicts with the desire to minimize insertion trauma mentioned above. Groups that follow this approach generally cite a study that determined the insertion tract wounds made by silicon probes of various sizes only affected acute tissue response, not the chronic response [144], to justify the attendant size increase caused by use of a shuttle [49].

The greatest difficulties in implementing a structural shuttle lie in the adhesion between the probe-shuttle couple. During implantation, it is essential to prevent sliding of the probe against the shuttle, which could lead to premature decoupling of the two and complicate probe placement. After the insertion is completed, the two must be decoupled without the probe accidentally retracting from the target of interest. Probe displacement is therefore one of the metrics of comparison between various shuttle solutions.

There are two main types of coupling methods: adjusting the surface of the shuttle to create material-material interactions that physically attract the shuttle to the probe or the use of biodissolvable adhesives to temporarily couple the two together during insertion. One advantage favoring biodissolvable adhesives over electrostatic interactions is that probe and shuttle dimensions are fully decoupled. Since the probe and shuttle are temporarily glued together, the dimensions of the shuttle need not exceed those of the probe in order for the probe not to get caught on surrounding tissue. Probes that are coupled to stiffeners electrostatically are removed by the addition of water, which migrates onto the hydrophilic surface and helps separate the two surfaces. Table 4 contains a summary of shuttle-based solutions that have been explored thus far. Other coupling methods include the use of a hold-and-release vacuum system [145].

### 5.3. Other Insertion Solutions

Some groups have developed other creative insertion solutions which include: the use of a material whose stiffness changes from stiff to compliant upon implantation [147] (for a full review of these materials, see [148]); coating a stiffer core with a thick polymer to attenuate mechanical mismatch, but retaining enough rigidity for penetration [101]; engineering the cross-section of a flexible probe to manipulate its bending moment without increasing cross-sectional area (with vertical stiffeners built into probe) [149]; adding a rigid tip to a flexible probe to enable penetration [150]; combining a rigid silicon tip with recording sites with a PEG-stiffened, flexible polyimide cable [151]; pre-penetrating the surface of the brain with a scalpel or 100 µm diameter tungsten wire [20]; using a biodissolvable brace to temporarily shorten probe length during insertion, thereby allowing for implantation of longer flexible probes without an insertion aid that penetrates the brain [152,153]; temporarily attaching of a silicon ultrasonic horn to enable probe insertion along its longitudinal resonance (however, this has only been performed with silicon probes to date [154]); fabricating a magnetic tip for a flexible probe to accelerate the probe through a magnetic field to its target in the brain [155].

## 6. Packaging

A complete probe system will interface between the biological application and the computer. Packaging plays the role of communicating signals from the microfabricated structures to low level processing before transmitting the signal to a computer, all within the confines of a hostile, biological environment. Below is a short survey of the various packaging techniques attempted in industry and research institutions.

**Figure 7:**
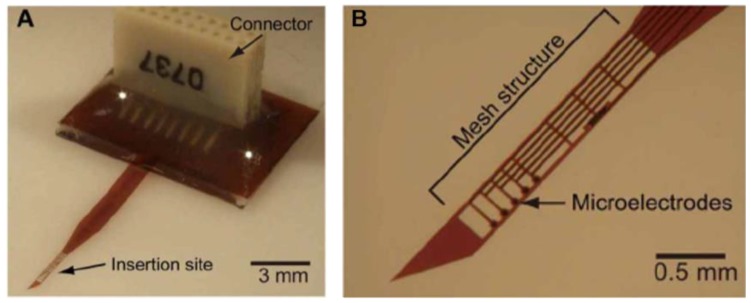
Example of a packaging solution for a polyimide intracortical probe. (**A**) Plastic plate bonded to backside of polyimide with epoxy to reinforce base during multiple reconnections. Omnetics connector soldered to gold pads and epoxy used as insulation; (**B**) Magnified view of polyimide-based, mesh, intracortical probe. Reproduced with permission from Creative Commons open access policy from [158].

Techniques connecting polymer-metal structures and processors include soldering, wire bonding, conductive epoxies, flip-chip bonding, and anisotropic conductive adhesives (ACAs). Soldering is a simple and well-known method of bonding two metals together by melting a third metal, typically lead-based, to form a joint. A largely manual process, soldering becomes more labor-intensive as MEMS structures become smaller and as the number of pads to solder increases. Furthermore, temperatures required for melting solder are typically higher than the degradation or melting temperature of the polymers previously discussed, with the exception of polyimide and OSTE+ [61]. Polyimide has been shown to be amenable to solder of various alloys [81,156,157] (see Figure 7 for an example of device packaging through soldering).

In integrated circuit (IC) fabrication, wire bonding is preferred for its scalability, automation, and repeatability, but it is difficult to use for polymer-based MEMS structures as the resonant frequencies of such structures lie within the range of frequencies of ultrasonic bonding [44]. Additionally, the thin metal film supported on a soft polymer is especially prone to damage from ultrasonic contact. Still, Meyer et al. have been successful in adapting wire bonding strategies in a “MicroFlex” technique for retinal implants wherein ball bonds are utilized as “rivets” to fasten a polyimide cable directly to a silicon chip, providing both a mechanical and electrical connection [159,160,161].

Another alternative is conductive epoxy [84,162]. Epoxies can be applied manually, but if batch processing is necessary, a precision pick-and-place robot or screen printing can be used [163]. For the proper alignment of adjoining surfaces, a pin and ring structure may be preferred over manual visual alignment [20,164]. Epoxies are useful due to their low curing temperatures and mechanical strength, but it is challenging to choose the appropriate viscosity [72,79], especially when approaching connections of higher density, as the risk of shorting increases.

Flip-chip bonding is the process of applying solder to bond pads of the chip, then aligning that chip upside-down onto the target, and finally reflowing the solder with heat [165]. As with soldering, the thermal budget must be considered when working with polymers, but flip-chip methods have already been successful in bonding silicon devices to polyimide cables [166,167]. For purely polymer systems, flip-chip bonding can be modified by replacing the solder with conductive materials processed at lower temperatures such as conductive epoxies [168], anisotropic conductive adhesives (ACAs), or even conductive polymers [169].

ACAs offer a number of advantages but are primarily preferred because they do not require precise alignment between device and chip. Metal-covered polymer spheres are distributed within the adhesive such that when compressed, the spheres contact and create a path of conduction. In this manner, the ACA is conductive only in the direction of compression and not in perpendicular directions. Wu et al. have successfully neural probes by incorporating a magnetic field to realign the spheres to create a conduction path [50]. Additionally, creating three-dimensional structures of stacked probes with ACAs is feasible [170]. Both the aforementioned devices joined features with over 100 µm pitch. In order to accommodate denser bond pads, especially for pitches lower than 50 µm, newer strategies such as double-layered film or insulated conductive particles may provide viable solutions [171]. Currently, ACAs for pitches as low as 10 µm are commercially available, but these typically require higher processing temperatures [172].

In addition to relaying signals from the microfabricated structure (i.e., electrodes on a neural probe), packaging plays the primary role of sealing the moisture-sensitive components from the aqueous, corrosive environment via encapsulation. Encapsulation of the device can be either hermetic (defined as keeping diffusion of helium lower than 10^−8^ cm^3^/s) or non-hermetic [166], and it can cover the entire device or only those portions that would otherwise fail if exposed to the in vivo environment [173]. Any gaps or voids in this seal could allow moisture to destroy the device either through leakage or condensation. Thus, in any implanted MEMS device, the electrically active bond pads must be entirely protected to prevent both mechanical and chemical damage. As USP Class VI polymers, PDMS and Parylene C are excellent candidates for choice of sealant for biomedical devices, but as organic materials, they can only provide non-hermetic sealing. For complete hermeticity, rigid housings such as titanium or ceramic may be more appropriate.

## 7. Conclusions

Flexible penetrating probes for neural stimulation and recording have grown tremendously as expressed by growth in the body of literature describing advances in novel microfabrication techniques, investigations on geometrical probe design, and innovative surgical placement solutions. Despite the decade long history, comprehensive guidance on polymer probe design and best practices on their implementation in vivo are still under development.

For continued progress, one major area that warrants special attention is the in vivo evaluation of probe geometry alterations and their effect on tissue-implant interface both acutely and chronically, and under representative recording or stimulation conditions. The benefit of using anchored structures for probes has not been evaluated histologically. Although minimizing probe footprint can attenuate the immune response, conflicting data prevent a definitive verdict leaving nagging questions on whether size differences, once beneath a certain threshold, dictate the stability of probe integration into surrounding tissue over time. Mechanically compliant substrate materials are being explored to further decrease the elastic modulus mismatch between the implant and brain, among other advantages. However, it is also not clear whether probes of varying stiffness, with a rigid region flanked by flexible regions [174], provide sufficient micromotion attenuation in the flexible regions surrounding electrode sites. Further evaluation of the long term biocompatibility of new materials being investigated for neural probes is also needed.

The compliant nature of polymer probes often necessitates the use of insertion aides, especially for deeper implants, to prevent buckling. Thus, long-term, single-neuron studies of deeper brain structures, such as the thalamus and hippocampus, are largely unexplored. Many flexible probe designs were limited to shank lengths of 1–3 mm, and their targets were cortical structures. Not only are longer probe shanks necessary to target deep brain structures, but, in moving from animal models to human models, the brain also scales in size and requires longer probes to access the relevant brain regions (Table 5). To achieve accurate placement, the selection of insertion method may vary according to species, location, age of the animal, and other study specific conditions. To minimize the size of the initial stab wound and maintain a tight connection between the implant and tissue, careful consideration of insertion methods that substantially increase the cross-section of the probe, even if temporary, is necessary. It is likely that approaches that favor minimal cross section may provide the greatest chronic interface stability.

While substantial progress has been made in realizing a wide variety of flexible penetrating probes, their in vivo evaluation is incomplete, preventing realization of their true potential as chronic neural interfaces. Addressing the remaining technical and biological challenges will pave the way to realizing the goal of long-term, stable, brain–machine interfaces for basic neuroscience and clinical therapy.

## Figures and Tables

**Table 1:** Young’s modulus comparison between analogous brain structures in various species.

Species	Young’s Modulus Hippocampus	Young’s Modulus Cerebellum	Young’s Modulus Cerebral Cortex	Young’s Modulus Dura Mater
Mouse	-	-	~7 kPa [11]	-
Rat	0.1 [12]–1.2 kPa [13]	0.3–0.45 kPa [14]	0.03–1.75 kPa [13]	0.4–1.2 MPa [15]
Human	-	-	-	32 MPa [16], 62 MPa [17]

**Table 2:** Properties of polymers for neural implants (silicon for reference). Values from Scholten [40] and Hassler [36] unless otherwise noted. PDMS: Polydimethylsiloxane; Parylene: Poly(p-xylylene); LCP: Liquid crystal polymer; BCB: Benzocyclobutene; USP: United States Pharmacopeia.

Property	Silicon	PDMS	Polyimide	Parylene C	SU-8	LCP	BCB
Young’s Modulus (GPa)	190	3.6 × 10^−4^–8.7 × 10^−4^	2.3–8.5	2.76	2.87–4.40 [41]	10.6	3.1 [42]
Melting Temperature (°C)	1414	-	-	290	-	280	-
Thermal Decomposition Temperature (°C)	-	~250	>550	-	300–315	-	-
Glass Transition Temperature (°C)	-	350 (oxidation); 750 (degradation)	325–410	90	200 [43]	-	>350 [42]
Degradation Temperature (°C)	-	250	510–620	125	380	-	-
Thermal conductivity (W/cm·K)	1.56 [44]	15–25	0.29	8.2	0.002–0.003	-	2.9 × 10^−3^
Dielectric Constant	11.9 [44]	2.6–3.8	3.5	3.1	3.2	3.0	2.65 [42]
Achievable Thicknesses (µm)	-	10–100 (spin coat)	1–15	1–100	1–300	25–3000	7–130
Biocompatibility	-	USP class VI	Yes (in vivo) [36,45]	USP class VI	Mild reactivity (in vivo) [36]	USP class VI	Yes (ex vivo) [46]

**Table 3:** Coatings used for flexible probes in literature.

Type of Coating	Ref.	Coating Method	Substrate Material	Increase in Cross Sectional Area	Increase in Buckling Force Threshold	Scale of Dissolution	Byproducts of Coating and Body’s Mechanism of Clearance
Silk	[104]	Dip-coating	Polyimide	N/A	With coating, sufficient to insert into fish brain	1–6 h anneal, dissolves in protease solution in 0.5–2.5 h	Peptides and amino acids, proteolytic degradation or assumed foreign body response [135]
	[90]	Layer-by-layer casting in PDMS	Polyimide	10 µm thick, with coating: to 70 or 1400 µm thick	0.04 mN, with 70 µm thick coating 12 mN and with 1400 µm thick coating 105 mN.	-	Peptides and amino acids, proteolytic degradation or assumed foreign body response [135]
	[126]	PDMS mold	Parylene-C	24 µm thick, with coating: 250 µm thick	2.6 mN, with coating: 300 mN	Days–weeks depending on duration of water annealing	Peptides and amino acids, proteolytic degradation or assumed foreign body response [135]
Polyethylene glycol (PEG)	[127]	Dip-coating	Parylene-C	6 µm thick, with coating: 11 µm thick	With coating, sufficient to insert into Biogel	-	Unknown [129]
	[8]	Pipetting PEG into Parylene channel	Parylene-C	10 µm thick, with coating: 20 µm thick	1 mN, with coating: 12 mN	200 s	Unknown [129]
	[126]	PDMS mold	Parylene-C	24 µm thick, with coating: 250 µm thick	2.6 mN, with coating: 47 mN	Within minutes, depending on molecular weight [8,75]	Unknown [129]
	[53]	PMMA mold	Milled gold leads coated with Parylene-C	12 µm thick, with coating: 137 µm thick	With coating, sufficient to insert into rat cortex	Within 3.5 h (gelatin mixture containing PEG)	Unknown [129] for PEG, gelatin breaks down into collagen, degraded by collagenase
Tyrosine-derived	[129]	Dip-coating	Polyimide	70 µm diameter, with coating: 180 µm diameter = 6.6x increase in cross-sectional area	With coating, sufficient to insert into agarose and parietal cortex of rat	20 min in vitro, in vivo recordings achieved within 60 min	Non-enzymatic, degraded by random hydrolytic chain cleavage [136,137]
	[128]	PDMS mold	SU-8	20 µm thick and 30 µm wide, with coating: 100 µm thick and 200 µm wide = 33x increase in cross-sectional area	50 mN with 200 µm thick coating	60 min in PBS, 120 min in agarose	Non-enzymatic, degraded by random hydrolytic chain cleavage [136,137]
Carboxy-methyl-cellulose (CMC)	[66]	Silicon and polyvinyl siloxane (PVS) mold	Parylene	2.7 µm thick and 10 µm wide, with coating: 135 µm thick, 100/300 µm wide = 500x–1500x increase in cross-sectional area	With coating, sufficient to insert into rat primary motor cortex	<3 min to become gel. Does not dissolve completely	Monosaccharides, dissolved in water [134]
	[134]	Spin-casting into silicon mold in centrifuge	No probe, only testing shuttle	N/A	With coating, sufficient to insert into rat motor cortex	Estimated 20 min, in-vivo took days	Monosaccharides, dissolved in water [134]
Sugars	[36]	Dip-coating	Polyimide	-	With coating, sufficient to insert into rat cortex	Dissolves immediately upon contact with cerebrospinal fluid	Monosaccharides, dissolved in water [134]
	[131]	Dip-coating	Benzocyclobutene (BCB)	-	With coating, buckles upon insertion into brain (species not included), mineral oil allowed penetration	Dissolves immediately upon contact with cerebrospinal fluid mineral oil helps delay dissolution	Monosaccharides, dissolved in water [134]
	[132]	Drawing lithography (allows for sharp tip)	Polyimide	10 µm wide and 10 µm thick, with coating: 40–300 µm added to each dimension	With coating increased up to 3.8 N	<100 s	Monosaccharides, dissolved in water [134]

**Table 4:** Shuttle-based solutions for stiffening flexible probes during insertion. SAM: Self-assembled monolayer; PEG: Polyethylene glycol.

Shuttle Type	Ref.	Coupling Method	Substrate Material	Increase in Cross Sectional Area	Increase in Buckling Force Threshold	Average Probe Displacement
Silicon, “Michigan styled“ neural probe as shuttle	[49]	Electronegative, self-assembled, carboxylic acid terminal monolayer	Polyimide, PDMS probes	Polyimide probe–125 µm thick, 196 wide; PDMS probe-100 µm thick, 200 µm wide. Shuttle added 15 µm of thickness and was 400 µm wide	With shuttle, sufficient to insert into motor cortex of rat	23 µm with SAM layer, 2365 µm without
Silicon backing	[82]	PEG adhesive with wicking channel on silicon stiffener and flip chip alignment	Polyimide	-	With shuttle, sufficient to insert into 0.6% agarose phantom and prefrontal cortex of rat	~28 µm
Nickel backing	[7,19]	Polyimide spin-coated onto electroplated Nickel, permanently attached stiffener	Polyimide	Polyimide probe 20 µm thick, Nickel backing 5 µm thick	With shuttle, sufficient to insert into rat cortex	N/A
Stainless steel microwire	[30]	PEG	Parylene C	Parylene encapsulated probe 20 µm thick, microwire 229 µm in diameter	With shuttle, sufficient to insert into rabbit cortex	N/A
Tungsten rod	[146]	None, aided by agarose block on top of brain	Polyimide	20 µm thick, 350 µm wide, tungsten rod adds diameter of 175 µm = 3.5x increase in cross-sectional area	With shuttle sufficient to insert into rat subthalamic nucleus	100 µm
Tungsten microwire	[64]	PEG	Parylene-C	Parylene probe 11 µm thick, tungsten microwire 250 µm thick	With shuttle sufficient to insert into rat cortex	90 µm

**Table 5:** Depths * of various brain targets across species.

Species and Reference	Hippocampus (mm)	Superior Colliculus (mm)	Substantia Nigra (mm)	Thalamus
Mouse [175,176]	1.7–5.3	0–6.3	4.0–5.9	2.3–5.2
Rat [177,178,179,180]	1.8–9.5	0.5–6.3	6.8–8.8	3.7–7.9
Monkey [181,182]	19.3–38.0	-	-	16.4–30.5
Human [179,183,184]	73.7–105.3	-	-	53.4–84.8

* Ranges represent the deeper value as compared between two atlases of the most superficial compared to the deepest aspect of the structure listed. All measurements taken with reference to the brain surface using Scalable Brain Atlas [185].
